# Inequalities that affect noncommunicable disease prevention and control 

**DOI:** 10.2471/BLT.26.297454

**Published:** 2026-11-01

**Authors:** Ahmad Reza Hosseinpoor, Nicole Bergen, Katherine Kirkby, Anne Schlotheuber, Leanne Riley, Virginia Arnold, Alarcos Cieza, Alain Labrique

**Affiliations:** a Data, Digital Health, Analytics and AI Department, World Health Organization, Avenue Appia 20, 1211 Geneva 27, Switzerland.; b Ottawa, Canada.; c Noncommunicable Diseases and Mental Health Department, World Health Organization, Geneva, Switzerland.; d Health Determinants, Promotion and Prevention Department, World Health Organization, Geneva, Switzerland.

Noncommunicable diseases such as cancers, cardiovascular diseases, chronic respiratory diseases and diabetes hinder the health and well-being of people and communities, place a heavy economic burden on health systems and societies and limit the development of human capital.^1^ Noncommunicable diseases disproportionately affect disadvantaged population groups, contributing to cycles of poverty and inequality. Tackling inequalities in these diseases and their underlying risk factors such as harmful use of alcohol, unhealthy diets, physical inactivity, tobacco use and air pollution is a key strategy for noncommunicable disease prevention and control.

Despite sustained global attention and high-level political commitments to addressing these diseases and their underlying risk factors, the world is largely off-track to meet global targets, including target 3.4 of the sustainable development goals (to reduce by one third premature mortality from noncommunicable diseases through prevention and treatment and promotion of mental health and well-being).^1^^,^^2^ While noncommunicable diseases were once considered to be an issue primarily for high-income countries,^3^^,^^4^ data from low- and middle-income countries over the past two decades demonstrate that these diseases are a concern in all countries.

The international health community needs to focus on the hidden inequalities in noncommunicable diseases to accelerate the prevention and treatment of diseases and the advancement of health equity. National averages of disease burden, risk factor prevalence and access to care may conceal differences by factors such as age, sex, economic status, education level, place of residence and disability status. Targeted actions to reduce within-country inequalities constitute an opportunity to make progress on addressing these diseases and their risk factors.

Strategies to address these inequalities benefit from a strong evidence base, including the use of inequality data to characterize the direction and magnitude of inequalities. A new World Health Organization (WHO) report, *State of inequality: noncommunicable diseases and their risk factors*,^5^ provides a comprehensive global assessment of country-level inequalities in noncommunicable diseases and their risk factors. Drawing from national data collected over the past decade, the report presents global trends in inequalities within noncommunicable disease burden, prevention and control.

Key findings from the report underscore that the poorest and least educated population groups within countries tended to have worse noncommunicable disease-related outcomes than the richest and most educated groups, but inequality patterns varied across indicators and contexts.^5^ Urban–rural inequalities were evident within countries, especially in health service coverage and environmental risk exposure. Additionally, in many settings, men and women had different risk factor prevalence and health service coverage.

The report highlights the importance of ensuring that population-wide noncommunicable disease strategies, such as the WHO best buys interventions,^6^ account for the needs of population groups that are falling behind. The integration of an equity lens into noncommunicable disease programming entails using targeted, evidence-informed interventions that address inequalities and their root causes.^7^ Evidence on within-country inequalities can help decision-makers identify population groups with the greatest needs and tailor existing, high-impact interventions to those groups. Addressing these inequalities also requires action beyond the health sector, through whole-of-government and whole-of-society approaches that tackle the social, economic, commercial and environmental determinants of health.

Data gaps constitute a critical barrier to effective policy-making, implementation and accountability. However, opportunities exist to strengthen the data by expanding, harmonizing and increasing the availability of disaggregated data. Disaggregated data should cover a wide array of indicators pertaining to unmet need, prevention, early detection, incidence, diagnosis, treatment, palliative care, survival and mortality, and cover diverse inequality dimensions. Inequality data that are comparable across countries allow for benchmarking and tracking progress towards global and regional goals.

Looking ahead, the strategic use of inequality data on noncommunicable diseases has great potential to contribute to the acceleration of prevention and control efforts. Three concrete next steps to enhance monitoring of the state of inequality in noncommunicable diseases and their risk factors are strengthening the collection and use of inequality data; expanding capacity for inequality monitoring at country, regional and global levels; and building on the report *State of inequality: noncommunicable diseases and their risk factors*^5^ through further inequality analyses, including at regional and country levels.
